# Extracellular Vesicles from Metastatic Rat Prostate Tumors Prime the Normal Prostate Tissue to Facilitate Tumor Growth

**DOI:** 10.1038/srep31805

**Published:** 2016-08-23

**Authors:** Sofia Halin Bergström, Christina Hägglöf, Elin Thysell, Anders Bergh, Pernilla Wikström, Marie Lundholm

**Affiliations:** 1 Department of Medical Biosciences, Pathology, Umeå University, Umeå, Sweden

## Abstract

Accumulating data indicates that tumor-derived extracellular vesicles (EVs) are responsible for tumor-promoting effects. However, if tumor EVs also prepare the tumor-bearing organ for subsequent tumor growth, and if this effect is different in low and high malignant tumors is not thoroughly explored. Here we used orthotopic rat Dunning R-3327 prostate tumors to compare the role of EVs from fast growing and metastatic MatLyLu (MLL) tumors with EVs from more indolent and non-metastatic Dunning G (G) tumors. Prostate tissue pre-conditioned with MLL-EVs *in vivo* facilitated G tumor establishment compared to G-EVs. MLL-EVs increased prostate epithelial proliferation and macrophage infiltration into the prostate compared to G-EVs. Both types of EVs increased macrophage endocytosis and the mRNA expression of genes associated with M2 polarization *in vitro*, with MLL-EVs giving the most pronounced effects. MLL-EVs also altered the mRNA expression of growth factors and cytokines in primary rat prostate fibroblasts compared to G-EVs, suggesting fibroblast activation. Our findings propose that EVs from metastatic tumors have the ability to prime the prostate tissue and enhance tumor growth to a higher extent than EVs from non-metastatic tumors. Identifying these differences could lead to novel therapeutic targets and potential prognostic markers for prostate cancer.

Neoplastic prostate cells interact with surrounding stromal cells, for instance smooth muscle cells, fibroblasts, immune cells, blood and lymph vessels, to create a tumor stroma that facilitates tumor growth and spread^1^,^2^. Such interactions also extend beyond the tumor stroma into the tumor-bearing organ, and adaptations such as blood and lymph vessel growth and influx of immune cells occurring far outside the tumor promote tumor growth in animal models of prostate cancer^3^,^4^,^5^,^6^,^7^. Similar changes are seen in the non-malignant prostate tissue in patients, for example a decrease of androgen receptors, an increase of phosphorylated EGF-receptor and PDGF-Rβ, influx of inflammatory cells, increased angiogenesis and altered extracellular matrix, and importantly these changes are related to tumor size, grade and patient outcome^7^. Hence, it is important to identify factors that mediate these influences to both the nearby tumor stroma and to more distant sites in the tumor-bearing organ, as increased knowledge of this could provide new therapeutic and prognostic tools for prostate cancer. Tumor-derived EVs have the potential to deliver such factors to recipient cells in both the nearby tumor stroma and also further away to the surrounding and distant organs.

Vesicles are released from most cell types, although more extensively from tumor cells as shown *in vitro* and by purification from plasma, ascites, and pleural effusions from cancer patients^8^,^9^,^10^. Cells can release various types of vesicles that based on size, biochemical properties or subcellular origin could affect their function. Two major types of vesicles have been described, large membrane vesicles (>200 nm) budding or shedding from the cell plasma membrane^11^,^12^ and endosomal-derived microvesicles (30–150 nm) called exosomes^13^,^14^. Moreover, tumor-derived microvesicles called large oncosomes (1–10 μm) have recently been implied to play a role in prostate cancer progression^15^,^16^. However, categorizing vesicles have been proven to be difficult and at present it is not known if one type of vesicle is biologically more important than another, so the term extracellular vesicles (EVs) is currently used by the field and in this study^17^.

The potential capacity of cancer-derived EVs to affect and modulate the tumor microenvironment and to create pre-metastatic niches in remote organs is receiving increasing interest. Recent studies show that EVs derived from cancer cells can differentiate fibroblasts into a more myofibroblastic phenotype with an increased potential to support tumor growth and angiogenesis^18^,^19^,^20^. Many studies have also shown that tumor-derived EVs are able to suppress tumor immunity^21^ and are important players in the formation of pre-metastatic niches^22^,^23^,^24^. Recently, we and others have shown that prostate cancer-derived exosomes down-regulate the activating receptor NKG2D on NK cells and CD8^+^ T cells, and that this most likely impairs lymphocyte cytotoxic function and promote tumor immune escape^25^,^26^. Moreover, there is evidence that exosomes derived from diverse cancer cell lines, including prostate cancer cells, selectively impair lymphocyte responses to interleukin-2^27^. Several studies have also been published supporting the role of exosomes as potential diagnostic and prognostic biomarkers for prostate cancer^28^,^29^,^30^,^31^. In addition, exosome-mediated drug resistance, including docetaxel-resistance in prostate cancer, has been studied and recognized as part of the cellular characteristics behind acquired treatment resistance^32^,^33^. Macrophages are closely linked to the chronic inflammatory processes during cancer progression^34^, however few studies have investigated the effect of tumor-secreted EVs on macrophages. Two recent studies have reported that EVs derived from breast^35^ and melanoma^36^ cancer cell lines stimulate macrophages resulting in increased NF-κB activity.

Accumulating data indicates that tumor-derived EVs are responsible for various tumor promoting effects, however the function of EVs derived from tumors with different characteristics is not thoroughly explored. As prostate tumors are known to have highly variable behavior it is of importance to evaluate how EVs from tumors with different aggressiveness may affect the surrounding microenvironment and the entire tumor-bearing organ, hence, affecting cancer progression.

In the present study we have examined: (1) if EVs from rat prostate tumors are able to prime the prostate microenvironment and in this way support subsequent prostate tumor growth *in vivo*, and (2) if EVs from tumors of different growth rates and metastatic capacity differ in this ability. We found that EVs derived from aggressive tumors primed the prostate tissue and subsequently promoted tumor growth compared to EVs from more indolent tumors. Furthermore, we show that tumor-derived EVs activated fibroblasts and modulated differentiated monocytes into M2-like macrophages, and EVs from aggressive tumors were more efficient in inducing these activated phenotypes.

## Results

### Isolation and Verification of Rat Prostate Tumor-Derived Extracellular Vesicles

**Figure 1:**
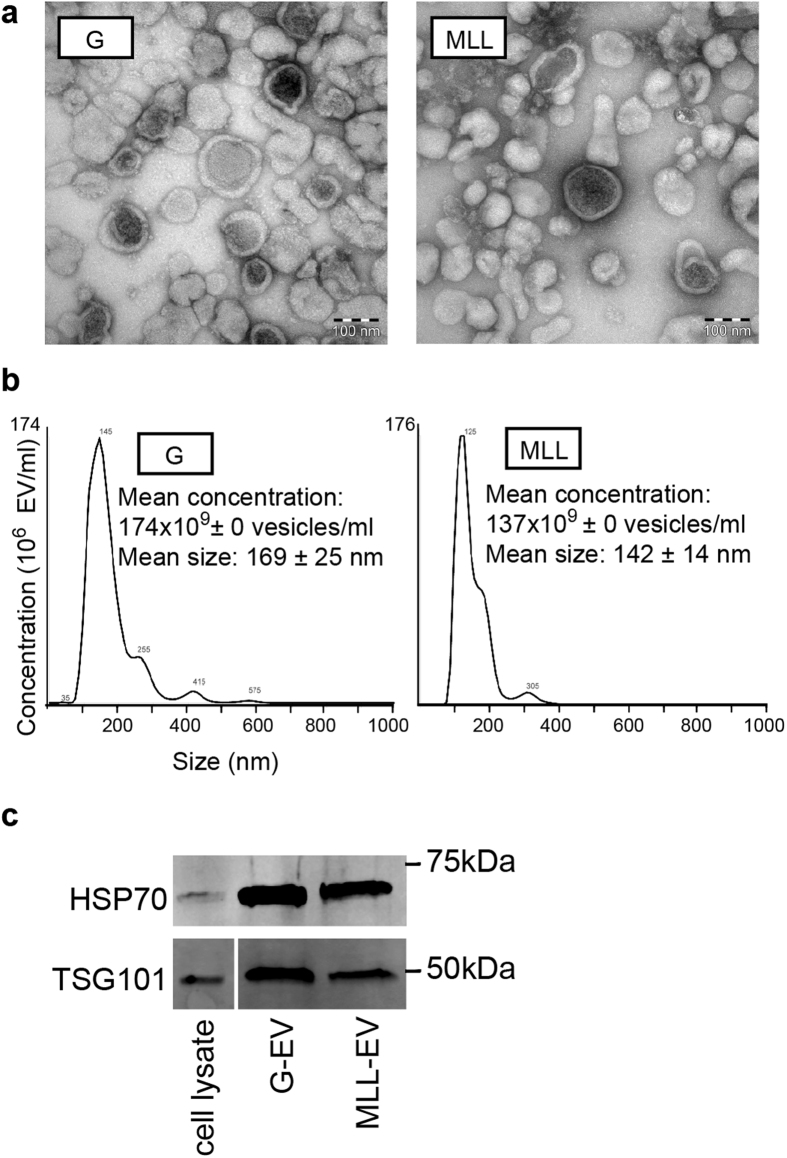
Characterization of tumor-derived extracellular vesicles (EVs). (**a**) Representative electron microscopic (EM) images of G- and MLL-EVs. Scale bar represents 100 nm. (**b**) Nanoparticle tracking analysis (NTA) of isolated EVs. The calculated size distribution (*n* = 3) and concentration (*n* = 3) are shown as a mean ± SD. The graphs shown are one measurement representative for G-EVs and MLL-EVs. (**c**) Representative Western Blot analyses of TSG101 and HSP70 in isolated G- and MLL-EVs. G tumor cell lysate was used as a cell control. Western Blots have been run under the same experimental conditions and were cropped to improve clarity.

To evaluate if prostate tumor-derived EVs were able to induce changes that were important for subsequent tumor growth in the non-malignant prostate we used the rat Dunning R-3327 prostate tumor model. This model consists of transplantable rat prostate cancer cell lines that are all derived from a spontaneous tumor in the dorso-lateral prostate of a Copenhagen rat^37^. Through *in vivo* passages, several tumor sublines with different characteristics have emerged and some of the sublines have also been established as *in vitro* cell lines. The cell lines can be injected back to fully immune competent Copenhagen rats to give tumors *in vivo*. In this study we used two of these cell lines, Dunning G (G) and MatLyLu (MLL). Both these cell lines form tumors that are anaplastic and low-differentiated with a poorly developed stroma^37^. The G tumor is slow-growing, androgen sensitive, and non-metastatic while the MLL tumors are rapidly growing, androgen insensitive, and metastatic. The tumors therefore represent different tumor grades^37^. G or MLL cells were injected orthotopically into the ventral prostate and EVs were isolated from established G tumors (*n* = 13) and MLL tumors (*n* = 17) using the standard sequential ultracentrifugation method^8^,^25^,^38^. To confirm and validate the presence of EVs, the EV fractions were evaluated by electron microscopy (EM), Nanoparticle tracking analysis (NTA) and Western Blot (WB) analysis. EM analysis of the EV fractions showed presence of a heterogeneous vesicle population and no cell debris (Fig. 1a). NTA (*n* = 3 in each group) demonstrated EVs ranging from 30 to 450 nm, the major part in between 80 and 200 nm (Fig. 1b), suggesting that the majority of the vesicles could be classified as exosomes^13^,^14^. There was no significant difference in quantity or mean particle size between G-EVs and MLL-EVs, though G-EVs showed slightly more particles of bigger size (Fig. 1b). The WB profile showed presence of the proteins Heat shock protein 70 (HSP70) and Tumor susceptibility gene 101 (TSG101), which are known exosome markers (ISEV guidelines^39^) (Fig. 1c).

### Prostates Pre-Conditioned with Tumor-Derived Extracellular Vesicles from Aggressive Tumors Facilitate Rat Prostate Tumor Growth

**Figure 2:**
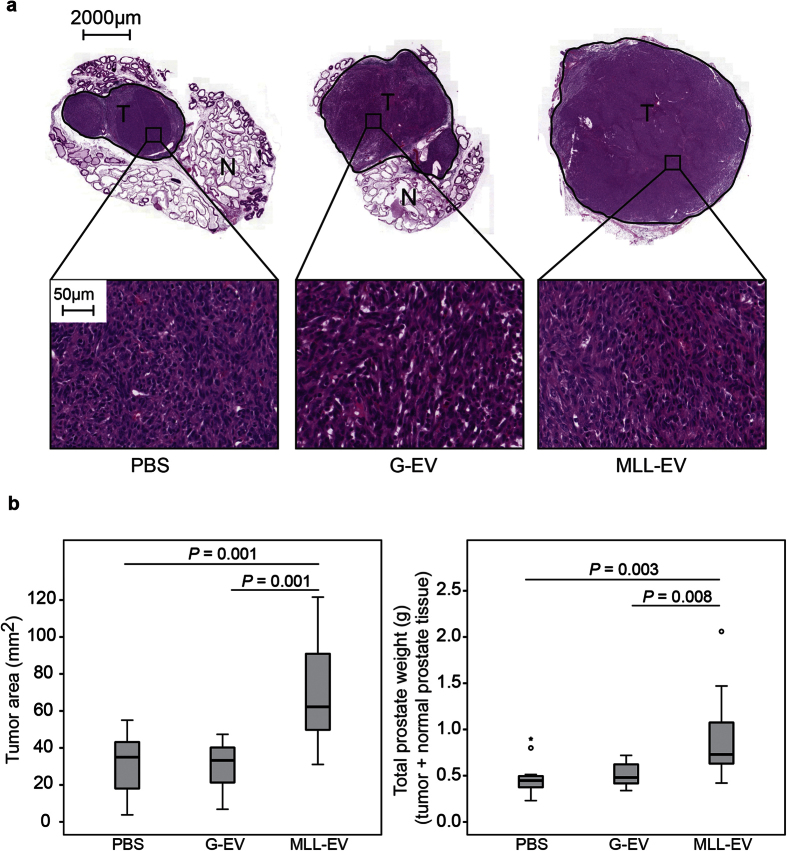
G tumor size. Rat ventral prostates were injected with MLL-EVs (*n* = 11), G-EVs (*n* = 11) or PBS (*n* = 13) as control and G tumor cells (1 × 10^5^ cells) were injected to the preconditioned prostates 72 hours later. (**a**) Representative sections of hematoxylin and eosin stained tumors at 8 weeks. T; tumor (encircled in black) and N; normal prostate tissue. Higher magnification shows a similar tumor morphology between the groups. (**b**) The largest tumor area (mm^2^) and the total prostate weight (tumor + normal prostate tissue, grams) were analyzed for each tumor at 8 weeks and each group was illustrated with box plots. Outlier values (o) and far-out values (*) are indicated.

To examine if prostate tumor-derived EVs could promote tumor establishment by inducing changes in the non-malignant prostate we injected MLL-EVs (*n* = 11), G-EVs (*n* = 11) or vehicle (PBS, *n* = 13) into the ventral prostate of naïve rats. As surgery was needed for injection into the prostate, one single injection was performed and the amount of exosomes injected (80 μg in 50 μl PBS) was approximately equal to the amount isolated from a half tumor. After 72 h, we injected G tumor cells (1 × 10^5^ cells) into the pre-conditioned prostates and analyzed tumor size eight weeks later. Interestingly, tumors established in prostates pre-conditioned with MLL-EVs were significantly larger compared to establishment in prostates pre-conditioned with G-EVs or PBS (Fig. 2a,b). This suggests that changes induced in the prostate tissue by EVs from aggressive tumors promote tumor growth compared to EVs from more indolent tumors. Tumors established in naïve (non-injected) prostates were not significantly different in size compared to tumors established in PBS injected prostates (data not shown). As the injection per se may cause a response in the prostate tissue, PBS injected prostates were chosen as appropriate controls throughout the study.

Analysis of the G tumors at sacrifice showed no significant differences in the general morphology of the tumors that were all anaplastic with a poorly developed stroma, showing that stromal cell content did not account for the increased tumor size in MLL-EVs conditioned prostates (Fig. 2a). Tumor cell proliferation (BrdU-labeling index) was not significantly different between the groups (PBS; 32.7 ± 3.1%, G-EVs; 36.6 ± 2.2% and MLL-EVs; 37.0 ± 2.1%, mean ± SEM, *P* = 0.74). This suggests that the tumor promoting effects of EVs from highly aggressive MLL tumors probably occurred early after tumor cell injection.

### Analysis of Rat Prostates Pre-Conditioned with Tumor-Derived Extracellular Vesicles

**Figure 3:**
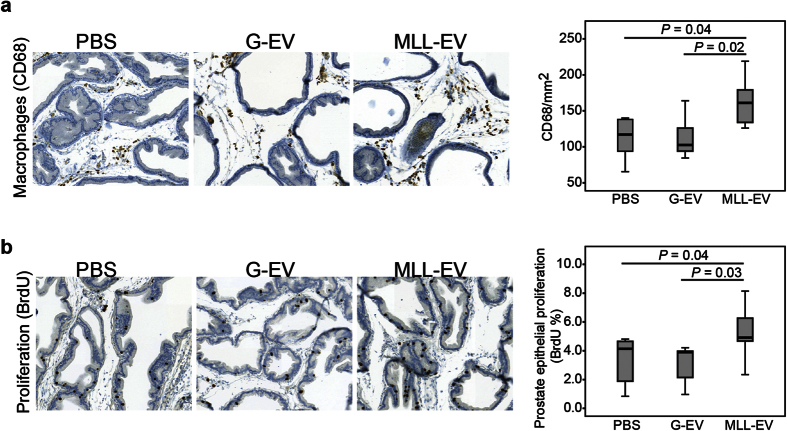
Immunostaining in rat prostates stimulated with tumor-derived extracellular vesicles (EVs). Rat ventral prostates were injected with PBS (*n* = 6), G-EVs (*n* = 6), or MLL-EVs (*n* = 6) and the prostate tissue was analyzed after 72 hours. Sections were immunostained and quantified for (**a**) macrophages (CD68) and (**b**) epithelial proliferation (BrdU). Sections show representative staining (brown) in each group (original magnification x200). Each group was illustrated with box plots.

To examine the effects of the tumor EVs in the prostate at the time of tumor cell injection, we evaluated prostate morphology 72 hours after tumor-derived EV injections (*n* = 6 in each group). The general morphology of the prostate tissue was similar between the groups (data not shown). We have recently shown that the gene expression in the tumor-adjacent rat prostate tissue is similar to a wound healing response and that macrophage density in this tissue is considerably higher in MLL tumors compared to G tumors^3^,^4^. In addition we have shown that macrophage-depletion in the tumor-bearing organ retarded tumor growth^5^. We therefore examined if there was a difference in macrophage density (CD68) in prostates conditioned with the different tumor EVs. Prostate tissue injected with MLL-EVs had significantly higher macrophage density than G-EVs injected prostates and PBS controls (Fig. 3a). This suggests that EVs from more aggressive tumors may prime the prostate tissue by increasing macrophage infiltration.

Although activated macrophages may have anti-tumor activity (proinflammatory, M1-like phenotype), tumor associated macrophages (TAM, alternative, M2-like phenotype) have been mainly shown to promote tumor progression^34^. We therefore further examined different macrophage markers (M1; Nitric oxide synthase 2 (NOS2) and M2; CD163 and Heme oxygenase 1 (HMOX1)) in prostates injected with the different tumor EVs. The density of NOS2 macrophages was higher in PBS injected controls (28.7 ± 2.8, mean ± SEM cells/mm^2^) compared to tumor-derived EV injected prostates (G-EVs; 19.4 ± 1.5, *P* = 0.02, MLL-EVs; 19.7 ± 3.5, mean ± SEM cells/mm^2^, *P* = 0.055), suggesting that tumor-derived EVs decrease the number of tumoricidal M1 macrophages. No significant differences were found in the quantity of HMOX1 or CD163 positive cells between the groups (data not shown), demonstrating that the tumor EVs had not affected the quantity of M2 macrophages in the pre-conditioned prostates at the time of tumor cell injection.

In addition, MLL-EVs increased prostate epithelial proliferation (BrdU-labeling) compared to the G-EVs and PBS (Fig. 3b), suggesting that EVs from more aggressive tumors could induce a higher proliferation in the normal prostate epithelium. This could be due to direct effects of the MLL-EVs on the prostate epithelium. The increased epithelial proliferation could, as in healing wounds, also be due to indirect paracrine effects from macrophages or other stromal cells activated by the MLL-EVs.

### Tumor-Derived Extracellular Vesicles Alter Cytokine Expression and Increase Endocytosis of Primary Rat Monocytes

**Figure 4:**
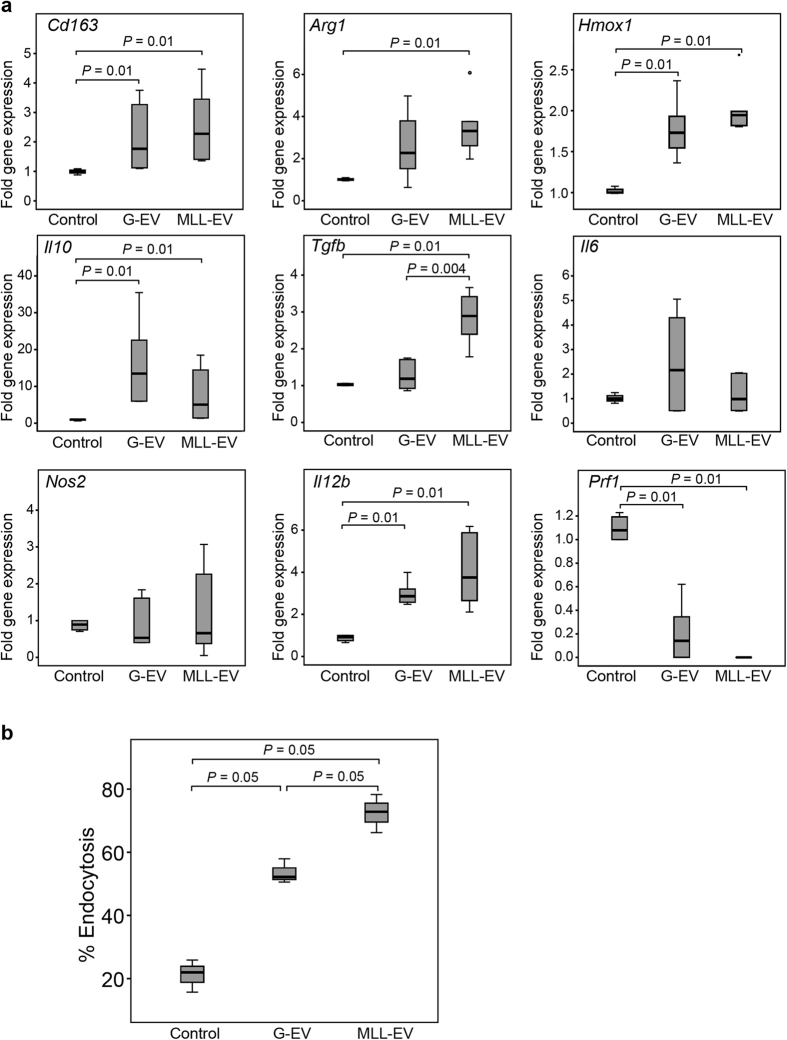
Phenotypic changes in EV-differentiated monocytes. (**a**) Expression of M1- or M2-associated genes in cultured monocytes with or without G-EVs or MLL-EVs was analyzed by RT-PCR. Shown is the fold gene expression from two independent experiments (*n* = 6 in each group), with control monocytes set as 1 and illustrated with box plots. Outlier values (o) are indicated. (**b**) Endocytosis by monocytes differentiated for 6 days with M-CSF in the presence or absence of G- or MLL-EVs was evaluated by measuring internalization of FITC-dextran followed by flow cytometry analysis. Shown is percentage endocytosis illustrated with box plots.

As the prostate tissue injected with MLL-EVs had significantly higher macrophage density than G-EVs and PBS injected prostates we further examined how the different tumor-derived EVs affected primary rat monocytes *in vitro*. Monocytes isolated from rat spleen were differentiated with Macrophage colony stimulating factor (M-CSF) for 6 days in the presence or absence of G-EVs or MLL-EVs. Macrophage markers, recognized to be generally of M1- or M2-like phenotype^34^, were analyzed by quantitative RT-PCR. The expression of the M2-associated markers *Cd163*, Arginase 1(*Arg1*), and *Hmox1* was significantly increased in monocytes/macrophages co-cultured with MLL-EVs compared to control monocytes but not compared to monocytes stimulated with G-EVs (Fig. 4a). The expression of *Cd163* and *Hmox1* were also significantly increased in monocytes stimulated with G-EVs compared to controls (Fig. 4a), suggesting that both types of tumor-EVs induced an M2-phenotype. The M2 typical anti-inflammatory cytokine Interleukin 10 (*Il10*) was also expressed at significantly higher levels in both G-EVs and MLL-EVs stimulated monocytes than in control monocytes (Fig. 4a). Moreover, the expression of Transforming growth factor beta (*Tgfβ*), a cytokine known to influence the microenvironment and promote tumor progression^40^, and to induce wound–healing responses was significantly higher in monocytes stimulated with MLL-EVs than G-EVs and controls (Fig. 4a). There was no difference in expression of the M1 macrophage proinflammatory cytokine Interleukin 6 (*Il6*) and the classical M1 marker *Nos2* between the differentially stimulated monocytes (Fig. 4a). On the other hand, expression of another proinflammatory cytokine, Interleukin 12 (*Il12*), was significantly upregulated in both MLL- and G-EV stimulated monocytes (Fig. 4a). Furthermore, the expression of Perforin 1 (*Prf1*) was highly decreased in monocytes stimulated with G-EVs and absent in monocytes stimulated in MLL-EVs compared to controls, suggestive of low cytolytic activity^41^.

Next we analyzed the endocytic function of the *in vitro* cultured monocytes. 6 days M-CSF *in vitro* differentiated monocytes were analyzed by flow cytometry for their ability to endocytose fluorescent-labeled dextran. The presence of tumor-derived EVs significantly increased the endocytic function of differentiated monocytes compared to controls (Fig. 4b), suggestive of a more M2-like macrophage phenotype^42^,^43^. Endocytosis was most increased in monocytes stimulated with the MLL-EVs.

Taken together this proposes that both types of tumor-derived EVs skew the monocytes into a more tumor promoting macrophage phenotype^34^. However, most factors examined did not differ in mRNA expression between monocytes stimulated with MLL-EVs compared to G-EVs, although MLL-EVs in general gave a more pronounced effect than the G-EVs when compared to controls.

### Prostate Tumor-Derived Extracellular Vesicles Activate Primary Rat Prostate Fibroblasts

Fibroblasts are important for tumor progression^2^ and it has been shown that tumor EVs can activate fibroblasts into myofibroblasts^18^,^19^,^20^. Activation of the prostate stroma could via paracrine signaling stimulate proliferation in both prostate epithelial cells (as seen in Fig. 3b) and in tumor cells. Furthermore, our previous study shows that the gene-expression pattern in tumor-adjacent non-malignant prostate is similar to that in wound healing suggesting that tumors activate the prostate stroma further away in the tumor-bearing organ^3^. We therefore explored if tumor-derived EVs could activate primary rat prostate fibroblasts *in vitro*.

Fibroblast activation was assessed with a rat wound healing gene expression RT-PCR array. Fibroblasts stimulated with both types of tumor-derived EVs had altered expression of several wound healing genes compared to PBS controls (Supporting information S1), suggesting that both types of EVs could activate fibroblasts. In addition, fibroblasts stimulated with MLL-EVs showed an induction of several factors known to be produced by prostate cancer associated fibroblasts^2^ compared to both G-EVs and PBS stimulation (Table 1). Among the genes with upregulated expression were growth factors such as Hepatocyte growth factor (*Hgf*) and Fibroblast growth factor 7 *(Fgf7)*, several cytokines like Colony stimulating factor 3 (*Csf3)*, C-X-C motif ligand 5 (*Cxcl5*)*, Il10, Il6*, and the matrix metalloproteinase 9 (*Mmp9*) (Table 1). Downregulated expression was shown for genes such as Smooth muscle alpha-actin (*Acta2)*, Connective tissue growth factor *(Ctgf)* and Transgelin (*Tagln)*.

*Cxcl5, Il10, Il6 and Hgf* were selected for confirmation with qRT-PCR in independently stimulated fibroblasts. Upregulation after MLL-EVs stimulation could be confirmed for *Il10* (vs. PBS: 2.6 fold*, P* = 0.01, *n* = 4; vs. G-EVs: 3.3 fold, *P* = 0.01, *n* = 4) and *Cxcl5* (vs. PBS: 13.2 fold, *P* = 0.01, n = 4; vs. G-EVs: 4.2 fold, *P* = 0.01, n = 4). However, the expression levels of *Il6* and *Hgf* were below detection limit (Ct value > 35).

Taken together, these results suggest that both types of EVs can activate rat prostate fibroblasts. However, MLL-EVs were more effective at inducing this activated phenotype which subsequently could support tumor cell growth and possibly also the proliferation of the normal epithelium (Fig. 3b).

### Extracellular Vesicles from Aggressive Tumors Increase Tumor Cell Viability *In Vitro*

**Figure 5:**
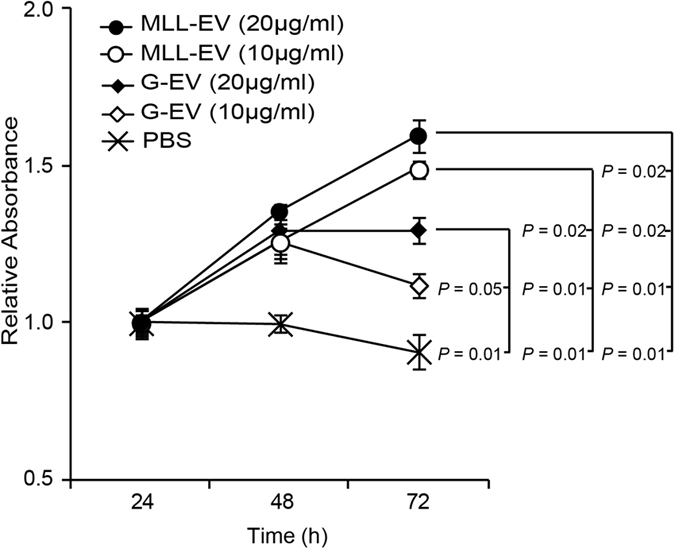
Cell viability of G tumor cells stimulated with tumor-derived EV *in vitro*. G tumor cells were cultured in serum-free medium and stimulated with PBS or different concentrations of G- or MLL-EVs and cell viability was measured at different time points using an *in vitro* MTT assay. Values are mean relative absorbance ± SEM compared to values at 24 hours.

The increased proliferation of non-malignant prostate epithelial cells *in vivo* (Fig. 3b) could also be due to direct effects of the MLL-EVs. If the MLL-EVs are present in the prostate at 72 hours, the tumor-derived EVs could thus in a similar manner also directly affect G tumor cell growth. To examine if the tumor-EVs directly affected G tumor cell growth we used an *in vitro* MTT viability assay under serum starvation. MLL-EVs increased G tumor cell viability over time, in a dose-dependent manner, compared to G-EVs and PBS, suggesting that the MLL-EVs also could have direct effects on tumor cell growth (Fig. 5). It is unknown if the tumor EVs were available for the tumor cells *in vivo* or if they are taken up by other cells in the prostate. Therefore, direct effects on tumor cell viability in the *in vivo* model cannot be excluded.

## Discussion

In order to survive and metastasize, tumor cells need to recruit and modulate non-malignant cells in the microenvironment^1^. Such adaptive changes reach not only the tumor adjacent stromal cells but also further away in the tumor-bearing organ^7^. In the Dunning rat tumor model, aggressive and highly metastatic tumors affect the tumor-bearing organ more intensively compared to more indolent tumors^4^ suggesting a difference in the tumor-host communication. Furthermore, alterations in the non-malignant epithelium and stroma in prostate cancer patients are associated with a poor prognosis^7^. This implies that changes not only in the tumor stroma but in the whole tumor-bearing organ are important for prostate tumor progression and increased understanding of the underlying mechanisms could thus lead to novel therapeutic targets and prognostic markers. Tumor cells have been shown to secrete EVs (microvesicles/exosomes) as a way of cell-cell communication^44^,^45^,^46^. Moreover, tumor EVs have been shown to activate cells in the nearby tumor stroma^47^ and also play important roles in the formation of pre-metastatic niches^22^,^23^,^24^. Importantly, here we show that tumor EVs also could be of central importance in the interactions with the tumor-bearing organ. Prostate tissue pre-conditioned with EVs from metastatic tumors (MLL-EVs) facilitated tumor establishment compared to EVs from non-metastatic tumors (G-EVs), indicating qualitative differences between tumor EVs from metastatic vs. non-metastatic tumors.

In this initial study the EVs were isolated from tumor tissue, since we consider them to best represent the *in vivo* situation. Some of the EVs are therefore most likely secreted from non-malignant cells such as inflammatory and stromal cells that possibly could contribute to the results. EVs secreted from mesenchymal stem cells and fibroblasts have been suggested to promote tumor growth^48^,^49^. Furthermore, a recent study shows differences in the microRNA (miRNA) content in exosomes from metastatic vs. non-metastatic pancreatic tumor cell lines, where miRNA from the metastasizing tumor prepares premetastatic niches^50^. Moreover, exosomes from high stage melanomas have been shown to have higher amounts of TYRP2, a melanoma-specific protein, than exosomes from low stage tumors^24^. This shows, together with the present study, that the content and functional importance of different types of EVs warrants further examinations.

We have previously shown that extratumoral macrophages promote tumor and vascular growth in a similar rat prostate model^5^ and our recent data also show that the normal prostate tissue adjacent to MLL tumors has considerably increased macrophage density compared to G tumors^4^. Furthermore, we have shown that the majority of infiltrating macrophages in prostate cancer patients have an M2 phenotype and are associated with poor patient prognosis^51^. We therefore examined if tumor-EVs had the capacity to attract macrophages to the normal prostate tissue. One injection of MLL-EVs significantly increased macrophage infiltration (CD68) to the normal prostate tissue *in vivo* compared to G-EVs and PBS injected controls, suggesting that EVs from metastatic tumors could increase macrophage infiltration to the normal tissue surrounding the tumor. Furthermore, both types of tumor-EVs induced a more tumor promoting macrophage phenotype *in vitro*, such as increased endocytosis and expression of TAM/M2 markers/cytokines and decreased cytolytic activity (*Prf1)*. This study therefore adds data to other studies also showing that exosomes derived from melanoma or breast cancer cells affect the cytokine and chemokine profile in macrophages^35^,^36^. Although MLL-EVs in general had a more pronounced effect than the G-EVs when compared to controls, *Tgfβ* was the only factor examined that was significantly changed in macrophages stimulated with MLL-EVs compared to G-EVs. TGFβ signaling plays important roles in wound healing and tumorigenesis^52^, for instance by suppressing inflammation and regulating immune cell functions which subsequently can drive tumor progression. As both types of tumor EVs induced a more tumor-promoting macrophage phenotype *in vitro*, the phenotype of infiltrating macrophages *in vivo* is therefore likely protumoral. As MLL-EVs increased the macrophage infiltration to the prostate compared to the G-EVs it could thus lead to increased number of protumoral macrophages that subsequently may enhance tumor establishment. However, no difference in the quantity of M2 markers (CD163 and HMOX1) was observed *in vivo*. This could be due to no effect of the injected tumor EVs on the recruited macrophages or shorter time duration *in vivo* (3 days) compared to the monocytes stimulation *in vitro* (6 days) which may result in less differentiated macrophages *in vivo*.

It has previously been shown that tumor EVs can activate fibroblasts into myofibroblasts^18^,^19^,^20^. In line with these studies, we show that prostate tumor derived EVs alter the expression of several wound healing genes indicating an activated fibroblast phenotype. In addition, this study indicates that there also is a difference in this ability between different types of tumor-EVs. Primary rat prostate fibroblasts stimulated with EVs from metastatic tumors had altered gene expression of several factors - related to fibroblast activation - that have been shown to affect prostate tumor progression and growth, such as *Hgf*^53^, *Mmp9*^54^, *Fgf7*^55^, and *Cxcl5*^56^ compared to EVs from non-metastatic tumors. As a result, this might contribute to the increased potential of the microenvironment to support tumor growth. It is also possible that activated fibroblasts induced the increased prostate epithelial proliferation observed in this model. Taken together, it is therefore important to further study the functional roles of monocytes and fibroblasts stimulated with tumor EVs.

In addition, as MLL-EVs increased viability of G tumor cells *in vitro* we cannot exclude that the effect on G tumor establishment *in vivo* was due to direct tumor-EV stimulation of the tumor cells. However, since other studies suggest rapid uptake of EVs from the circulation and by cells *in vitro*^24^,^50^ it is more likely that the effect seen on tumor growth is because of EV-induced microenvironment changes contributing to an important rate-limiting step in tumor establishment. Furthermore, the precise site of injection can differ between the injected EVs and the tumor cells, making direct influences of the EVs on the tumor cells less likely.

In summary we here show that normal rat prostate tissue pre-conditioned with EVs from metastatic rat prostate tumors enhanced tumor establishment of tumor cells injected 72 hours later compared to prostate tissue pre-conditioned with EVs from non-metastatic tumors. This could be due to increased infiltration of macrophages to the prostate tissue and induction of a more tumor-promoting macrophage phenotype by the tumor-EVs. In addition, EVs from metastatic tumors may activate fibroblasts into a more cancer-promoting phenotype. This suggests that EVs from aggressive and metastatic tumors affect not only the close by tumor stroma but also the whole tumor-bearing organ to facilitate tumor progression. Further studies to examine the EV content from aggressive compared to non-aggressive tumors are highly important as this could potentially lead to novel prognostic markers and therapeutic targets for prostate cancer.

## Materials and Methods

### Cell Culture

Dunning G (G) and MatLyLu (MLL) rat prostate tumor cells were ordered from European Collection of Cell Cultures (ECACC) and were grown in culture as previously described^37^.

### Orthotopic G and MLL Tumors for Preparation of Extracellular Vesicles

Three to four month old, fully immune competent, male Copenhagen rats (Charles River, bred at our animal facility) were used in all animal studies. Animal experiments were carried out in accordance with protocols approved by the Umeå Ethical Committee for animal studies (permit number A110-12).

Animals were anaesthetized and MLL cells (4 × 10^3^ cells in 10 μl RPMI, *n* = *17*) or G cells (2 × 10^5^ in 10 μl RPMI, *n* = *13*) were injected into the ventral prostate as previously described^57^. Each animal had a single tumor and when the tumor types were of similar size (*P* = 0.25) the MLL (14 days, mean tumor weigh ± SEM, 3.5 ± 0.4 g) and G (8 weeks, 4.1 ± 0.4 g) tumors were removed, weighed and used for tumor EV isolation.

### Isolation and Evaluation of Rat Prostate Tumor-Derived Extracellular Vesicles

Non-necrotic MLL or G tumor tissue was cut into small pieces (2–3 mm^2^) and placed in RPMI with 1 mg/ml Collagenase type I (Sigma-Aldrich) for 45 min at 37 °C to digest the tissue^58^. The cell suspension was filtrated through a 70 μm cell strainer and EVs were isolated by standard ultracentrifugation as previously described by us and others^8^,^25^,^38^. Briefly, cell suspension was cleared of cells and debris by sequential centrifugations at 3,000 *g* for 30 min and 10,000 *g* for 35 min at 4 °C. The pellet was discarded and the supernatant was passed through a 0.22-μm filter and ultracentrifuged at 110,000 *g* for 2 h. The EV pellet was loaded on a 20–40% sucrose gradient and the ultracentrifugation step was repeated. The EVs captured in the sucrose layer were collected and washed with PBS. The EVs were resuspended in PBS and the protein concentration was determined using the BCA protein assay (Thermo Scientific -Pierce).

To confirm presence of EVs, electron microscopy (EM) was performed at the electron microscopy unit Emil, Clinical Research Center, Huddinge, Sweden. Number and size distribution of isolated EVs were evaluated by Nanoparticle tracking analysis (NTA) (NanoSight N300, Malvern Instruments Ltd.), *n* = 3 in each group. Western blot analysis was performed to determine presence of EV markers. Briefly, samples (12 μg protein) were separated by 5–15% Mini-PROTEAN TXG gels (Bio-Rad) and subsequently transferred to PVDF membranes (Mini format, Bio-Rad). Membranes were blocked in Odyssey Blocking buffer (LI-COR Biosciences) before being incubated with primary antibodies anti-TSG101 (clone 4A10, Abcam) or anti-HSP70 (ab3148, Abcam) overnight at 4 °C. After washing in PBST, secondary antibodies (Invitrogen, diluted 1:20,000 in 1% PBST) were applied and incubated for 1 h at RT. After washing with PBST, the proteins were visualized using LI-COR ODYSSEY CLx.

### Pre-Conditioning of Rat Ventral Prostate with Tumor-Derived Extracellular Vesicles

Rats were anesthetized and a surgical incision was made in the lower abdomen to gain access to the prostate. G-EVs, MLL-EVs (80 μg in 50 μl PBS) or PBS only were injected into the ventral prostate. After 72 h, animals were sacrificed, the prostate tissue removed, formalin fixed and paraffin embedded (*n* = 6 in each group). Some of the animals (*n* = 11–13 in each group, two independent experiments) were injected with G tumor cells (1 × 10^5^ cells in 10 μl RPMI) into the pre-conditioned ventral prostate and tumors were allowed to grow for 8 weeks before the animals were sacrificed. One hour prior to sacrifice the animals were injected intraperitoneal with bromodeoxyuridine (BrdU, 50 mg/kg, Sigma-Aldrich) to label proliferating cells and then prostates containing the tumors were removed, weighed and formalin fixed. Each animal had established a single spherical tumor.

### Morphological Analysis

Immunohistochemistry was essentially performed as previously described^5^,^59^ with primary antibodies against BrdU (BD Biosciences, #347580, 1:200), CD68 (Serotec, #MCA 341R, 1:200), HMOX1 (Enzo stressgen, #ADI-SPA-895, 1:100), CD163 (Serotec, #MCA 342R, 1:100), and NOS2 (Abcam, #Ab15323, 1:400). Rat prostate tissue sections were stained using Ventana Benchmark Ultra (Ventana Medical Systems Inc.) with CC1 as antigen retrieval.

The number of CD68, CD163, NOS2, and HMOX1 positive cells per mm^2^ was assessed in 5–10 randomly chosen areas (0.2–1 mm^2^/area) in each animal using Pannoramic Viewer software version 1.15 (3DHistech, www.3dhistech.com). The fraction of BrdU positive cells was measured in approximately 1000 prostate epithelial cells.

Tumor size was analyzed both as wet weight and by morphological measurements of the tumor area. Total prostate wet weight (tumor + normal prostate tissue) was measured directly after sacrifice and used to represent tumor size (g) for each animal. As wet weight was both normal prostate tissue and tumor tissue we also examined the tumor area. Tumors were sectioned at different levels and the sections were haematoxylin- and eosin-stained and analyzed with the Pannoramic Viewer (3DHistech) to locate the section with the largest tumor area. The largest tumor area (mm^2^) was chosen to represent tumor size for each animal.

### Tumor-derived Extracellular Vesicle Stimulation of Primary Rat Prostate Fibroblasts

Primary rat mesenchymal cell cultures, here referred to as fibroblasts, were established and maintained according to Tuxhorn *et al*.^60^. Rat ventral prostate tissues were cut into small tissue pieces and washed with DMEM (Gibco). The tissue pieces were then placed into 24-well cell culture plates (Thermo scientific) and provided with DMEM supplemented with 5% NU serum (Corning, Thermo scientific), 5% FBS, 10 nM dihydrotestosterone (Sigma-Aldrich), 5 μg/ml insulin (Sigma-Aldrich) and 2 mM penicillin/streptomycin (Gibco). The tissues were then cultivated in a cell incubator until stromal cells migrated out of the tissue pieces and reached confluence. The remaining tissue was removed and the cells were maintained using standard cell culture procedures. Verification of a mesenchymal phenotype was done by immunohistochemical staining; showing that the cells were vimentin-positive (Atlas, HPA001762) and cytokeratin-5 and -18 negative (CK5, Covance, AF138 and CK18 Progen, GP-CK18). For EV-stimulation, the primary rat fibroblast cultures were seeded into 24 well plates (1.5 × 10^4^ cells/well) and starved over night. The cells were then stimulated with G-EVs (100 μg/ml), MLL- EVs (100 μg/ml) or equal volume of PBS for 72 h (*n* = 4 wells in each group)^18^.

### Monocyte Isolation and Stimulation with Tumor-Derived Extracellular Vesicles

Cell suspensions were prepared from spleens of 7–9 week old rats in Hank’s BSS (Gibco). Mononuclear cells were isolated from cell suspension by gradient centrifugation on Lymphoprep (Nycomed). Monocytes were further purified by magnetic-activated cell sorting (MACS) using anti-CD11b:Biotin (clone OX-42; Nordicbiosite) with anti-Biotin microbeads (Miltenyi Biotec) according to the manufacturer’s instructions, resulting in a monocyte population of >95% purity (data not shown). Monocytes were cultured as previously described^42^ and differentiated with M-CSF 50 ng/ml (Sigma-Aldrich). MLL- or G-EVs (30 μg protein/10^6^ monocytes)^10^ were added to the culture, the medium was changed at day 3 and at day 6 the cells were harvested and subjected to further analysis^42^.

### Endocytosis

To examine endocytic function of the cultured monocytes differentiated for 6 days in the presence or absence of EVs, FITC-dextran internalization was measured as previously described^42^. Briefly, macrophages were harvested, the cell numbers were adjusted to 5 × 10^5^ cells/ml of RPMI medium, and 1 ml was seeded per well in a 24-well plate (*n* = 3 wells/group). Macrophages were pre-incubated on ice for 30 minutes and then incubated with 20 μg/ml FITC-dextran (Sigma-Aldrich) for 15 min at 37 °C. Incubation at 4 °C was used as a control to exclude unspecific cell surface binding. The cells were washed three times using 1 ml of cold PBS containing 5% FBS and percentage endocytosis (FITC-positive cells) was determined by flow cytometry (FACSCalibur; BD Biosciences FACS) and analyzed using CellQuest software (BD Biosciences).

### RNA Preparation

RNA was isolated with the RNeasy plus mini kit (Qiagen) according to the manufacturer’s instructions and quantified using a Nanodrop spectrophotometer (Thermo scientific). The integrity of the RNA was determined using an Agilent 2100 BioAnalyzer (Agilent).

### qRT-PCR Array

After stimulation of primary rat fibroblasts, the RNA was isolated and 300 ng of RNA from each treatment group was converted to cDNA with the RT^2^ First strand kit (Qiagen). Gene expression profiles were analyzed with the Rat Wound Healing PCR Array (PARN-121ZA; Qiagen) according to the manufacturer’s instructions. The resulting data (fold changes in Ct values of all genes) was analyzed with the PCR array data analysis web portal (Qiagen) with the average Ct value of the housekeeping genes *Rpl13a, Ldha* and *Actb* used as reference.

### qRT-PCR Analysis

For verification of selected genes, total RNA was isolated and cDNA was prepared using the Superscript VILO cDNA Synthesis kit (Invitrogen). Quantitect Primer Assays (Qiagen) were used for analyzing expression of *Cd163*, *Il6, Il10, Il12*, *Nos2*, *Arg1*, *Tgfβ*, *Hgf*, *Prf1* and *Cxcl5*. TaqMan gene expression assay (Applied Biosystems) was used to measure expression of *Hmox1* (Rn 01536933_m1). The RT-PCR-reactions were run on a TaqMan 7900HT (Applied Biosystems, Life Technologies). Relative gene expression was determined using the comparative Ct method, with *Rpl13* (Thermo Scientific) as a standard reference gene for primer assays and *Actb* (Rn 00667869_m1) for gene expression assay, as described in the Applied Biosystems user bulletin^61^.

### MTT viability assay

Viability was determined by the MTT assay (Roche Diagnostics). Briefly, G cells (6 × 10^3^ cells/well) were seeded in 100 μl of complete medium in a 96 well plate and incubated in 37 °C over night. The following day, cells were carefully washed with PBS and incubated in serum free medium for 6 h, washed again and incubated with different concentrations of G- and MLL-EVs in serum free media for 24–72 hours. PBS was used as control. MTT labeling reagent and solubilization solution was added at the different time-points and absorbance measured as previously described^59^.

### Statistical Analysis

Statistical analysis was performed using SPSS Statistics 22 (SPSS Inc.). The non-parametric Kruskal-Wallis *H* test was used for multiple comparisons. The non-parametric Mann-Whitney *U* test was used when comparing two groups. A *P*-value < 0.05 (two-sided) was considered statistically significant.

## Additional Information

**How to cite this article**: Bergström, S. H. *et al*. Extracellular Vesicles from Metastatic Rat Prostate Tumors Prime the Normal Prostate Tissue to Facilitate Tumor Growth. *Sci. Rep*. **6**, 31805; doi: 10.1038/srep31805 (2016).

## Supplementary Material

Supplementary Information

## Figures and Tables

**Table 1:** RT-PCR wound healing array of primary rat fibroblasts stimulated with tumor-derived extracellular vesicles.

Gene Symbol	Description	Fold-change	
		MLL-EVs vs. G EVs	MLL-EVs vs. PBS
*Csf3*	Colony stimulating factor 3	10.3	5.3
*Cxcl3*	Chemokine ligand 3	6.4	23.4
*Cxcl5*	Chemokine ligand 5	47.0	34.8
*Fgf7*	Fibroblast growth factor 7	6.0	8.8
*Hgf*	Hepatocyte growth factor	5.2	13.8
*Il10*	Interleukin 10	5.1	1.3
*Il6*	Interleukin 6	8.2	24.1
*Mmp9*	Matrix metallopeptidase 9	6.6	14.4
*Acta2*	Smooth muscle alpha-actin	−4.4	−2.1
*Col5a3*	Collagen, type V, alpha 3	−3.3	−2.4
*Ctgf*	Connective tissue growth factor	−3.4	−2.6
*Itga4*	Integrin, alpha 4	−3.0	−1.7
*Tagln*	Transgelin	−3.7	−1.6

Genes differentially expressed in fibroblasts stimulated with MLL extracellular vesicles (MLL-EVs) compared to G-EVs or PBS (≥3-fold up- or downregulation with at least one Ct value below 30). See Supplementary Table S1 for complete data set.
