# Supplementary material for: Extracellular Vesicles from Metastatic Rat Prostate Tumors Prime the Normal Prostate Tissue to Facilitate Tumor Growth
Source: Sci Rep. 2016 Aug 23;6:31805. doi: 10.1038/srep31805 (PMC4994101; doi:10.1038/srep31805)
Supplement: Supplementary Information [file srep31805-s1.doc]

**Extracellular Vesicles from Metastatic Rat Prostate Tumors Prime the Normal Prostate Tissue to Facilitate Tumor Growth**

Sofia Halin Bergström, Christina Hägglöf, Elin Thysell, Anders Bergh, Pernilla Wikström, and Marie Lundholm*

Department of Medical Biosciences, Pathology, Umeå University, Umeå, Sweden

***Corresponding author**: Marie Lundholm, Department of Medical Biosciences, Pathology, 6M, second floor, Umeå University, SE-90185, Umeå, Sweden. E-mail: [marie.lundholm@umu.se](mailto:marie.lundholm@umu.se); Phone: +46 90 785 44 05, Fax: +46 90 785 44 84

| **Supplementary Table S1.** Wound healing RT-PCR array showing gene expression in primary fibroblasts stimulated with PBS, G-EV and MLL-EV for 72 hours. | | | |
| --- | --- | --- | --- |
| **Gene symbol** | **PBS** | **G-EV** | **MLL-EV** |
| *Acta2* | 7,1E+00 | 1,5E+01 | 3,4E+00 |
| *Actc1* | 2,4E-04 | 1,2E-04 | 1,8E-04 |
| *Angpt1* | 2,0E-02 | 1,1E-01 | 1,7E-01 |
| *Ccl12* | 1,9E-03 | 9,3E-03 | 7,2E-03 |
| *Ccl7* | 1,6E+00 | 3,3E+00 | 8,4E+00 |
| *Cd40lg* | 1,7E-04 | 2,3E-04 | 1,8E-04 |
| *Cdh1* | 7,3E-03 | 5,2E-04 | 1,8E-03 |
| *Col14a1* | 2,8E-02 | 8,6E-02 | 9,8E-02 |
| *Col1a1* | 8,2E+00 | 1,3E+01 | 4,4E+00 |
| *Col1a2* | 1,0E+01 | 2,8E+04 | 9,5E+00 |
| *Col3a1* | 3,8E+00 | 6,5E+00 | 2,4E+00 |
| *Col4a1* | 7,1E-01 | 2,2E+00 | 1,1E+00 |
| *Col4a3* | 3,3E-02 | 2,0E-02 | 2,8E-02 |
| *Col5a1* | 1,9E-01 | 6,4E-01 | 2,5E-01 |
| *Col5a2* | 1,2E+00 | 3,3E+00 | 1,2E+00 |
| *Col5a3* | 8,1E-03 | 1,1E-02 | 3,5E-03 |
| *Csf2* | 2,5E-03 | 4,3E-03 | 1,2E-02 |
| *Csf3* | 1,4E-03 | 7,1E-04 | 7,3E-03 |
| *Ctgf* | 1,6E+00 | 2,2E+00 | 6,5E-01 |
| *Ctnnb1* | 1,5E-01 | 5,7E-01 | 5,2E-01 |
| *Ctsg* | 3,9E-04 | 1,2E-04 | 1,8E-04 |
| *Ctsk* | 7,7E-02 | 1,2E-01 | 9,7E-02 |
| *Ctsl1* | 2,7E-01 | 9,3E-01 | 1,1E+00 |
| *Cxcl1* | 1,2E-02 | 2,6E-02 | 3,6E+05 |
| *Cxcl11* | 1,7E-04 | 2,7E-04 | 1,8E-03 |
| *Cxcl3* | 1,1E-03 | 4,1E-03 | 2,6E-02 |
| *Cxcl5* | 1,7E-04 | 1,2E-04 | 5,8E-03 |
| *Egf* | 6,7E-04 | 1,3E-03 | 1,1E-03 |
| *Egfr* | 3,1E-02 | 4,9E-02 | 7,0E-02 |
| *F13a1* | 1,0E-03 | 3,6E-04 | 2,4E-04 |
| *F3* | 3,1E-01 | 1,1E+00 | 3,7E-01 |
| *Fga* | 1,2E-03 | 1,2E-04 | 3,7E-04 |
| *Fgf10* | 2,8E-02 | 8,3E-02 | 1,2E-01 |
| *Fgf2* | 2,1E-03 | 9,3E-02 | 5,8E-02 |
| *Fgf7* | 5,3E-02 | 7,8E-02 | 4,6E-01 |
| *Hbegf* | 1,3E-02 | 4,6E-02 | 2,7E-02 |
| *Hgf* | 4,5E-03 | 1,2E-02 | 6,3E-02 |
| *Ifng* | 1,7E-04 | 1,2E-04 | 1,8E-04 |
| *Igf1* | 2,6E-02 | 3,8E-02 | 8,0E-02 |
| *Il10* | 9,5E-03 | 2,5E-03 | 1,3E-02 |
| *Il1b* | 1,7E-03 | 8,6E-04 | 3,0E-03 |
| *Il2* | 7,9E-04 | 1,2E-04 | 1,9E-04 |
| *Il4* | 1,7E-04 | 1,2E-04 | 1,8E-04 |
| *Il6* | 8,1E-02 | 2,4E-01 | 1,9E+00 |
| *Il6st* | 1,3E-01 | 2,4E-01 | 3,1E-01 |
| *Itga1* | 5,0E-02 | 2,5E-01 | 1,8E-01 |
| *Itga2* | 1,4E-03 | 9,7E-03 | 1,3E-02 |
| *Itga3* | 1,8E-02 | 1,1E-01 | 6,7E-02 |
| *Itga4* | 4,6E-03 | 8,0E-03 | 2,6E-03 |
| *Itga5* | 2,8E-02 | 8,8E-02 | 6,1E-02 |
| *Itga6* | 1,9E-02 | 2,0E-04 | 5,7E-04 |
| *Itgav* | 3,5E-01 | 5,7E-01 | 3,8E-01 |
| *Itgb1* | 5,9E-01 | 2,0E+00 | 1,1E+00 |
| *Itgb3* | 7,3E-02 | 2,4E-02 | 2,1E-02 |
| *Itgb5* | 9,2E-02 | 3,2E-01 | 2,3E-01 |
| *Itgb6* | 1,7E-03 | 5,6E-04 | 1,8E-03 |
| *Mapk1* | 9,7E-02 | 2,2E-01 | 1,9E-01 |
| *Mapk3* | 5,5E-02 | 1,4E-01 | 1,2E-01 |
| *Mif* | 4,3E-01 | 5,2E-01 | 6,9E-01 |
| *Mmp1a* | 2,5E-04 | 1,2E-04 | 5,5E-04 |
| *Mmp2* | 1,1E+00 | 1,4E+00 | 2,0E+00 |
| *Mmp7* | 1,7E-04 | 1,4E-04 | 7,2E-04 |
| *Mmp9* | 4,5E-04 | 9,8E-04 | 6,5E-03 |
| *Pdgfa* | 1,5E-02 | 1,4E-01 | 6,5E-02 |
| *Plat* | 3,4E-02 | 1,0E-01 | 1,1E-01 |
| *Plau* | 7,9E-03 | 2,2E-02 | 9,5E-03 |
| *Plaur* | 2,2E-02 | 1,7E-02 | 1,2E-02 |
| *Plg* | 1,1E-03 | 4,6E-04 | 3,2E-04 |
| *Pten* | 6,9E-02 | 1,8E-01 | 1,6E-01 |
| *Ptgs2* | 3,6E-02 | 4,4E-01 | 8,0E-01 |
| *Rac1* | 2,7E-01 | 6,9E-01 | 6,0E-01 |
| *Rhoa* | 3,7E-01 | 1,0E+00 | 7,8E-01 |
| *Serpine1* | 5,7E-01 | 2,6E+00 | 1,2E+00 |
| *Stat3* | 3,2E-02 | 3,2E-01 | 3,1E-01 |
| *Tagln* | 4,1E+00 | 9,5E+00 | 2,5E+00 |
| *Tgfa* | 9,1E-03 | 2,8E-02 | 2,3E-02 |
| *Tgfb1* | 8,4E-02 | 2,0E-01 | 1,4E-01 |
| *Tgfbr3* | 3,3E-02 | 4,3E-02 | 1,1E-01 |
| *Timp1* | 2,4E+00 | 5,0E+00 | 8,4E+00 |
| *Tnf* | 1,7E-04 | 3,4E-04 | 4,1E-04 |
| *Vegfa* | 4,3E-02 | 2,2E-01 | 2,4E-01 |
| *Vtn* | 4,3E-04 | 2,3E-04 | 1,8E-04 |
| *Wisp1* | 8,3E-02 | 3,0E-01 | 1,3E-01 |
| *Wnt5a* | 1,4E-02 | 4,0E-02 | 7,8E-02 |
| Normalized gene expression according to the 2^ΔCt (Ct(GOI) – Ave Ct(HKG)) data analysis method. GOI; Gene of interest, HKG; House keeping gene | | | |
